# Editorial: Impulse Control Disorders, Impulsivity and Related Behaviors in Parkinson's Disease

**DOI:** 10.3389/fneur.2019.00972

**Published:** 2019-09-11

**Authors:** Mayela Rodríguez-Violante, Angelo Antonini

**Affiliations:** ^1^Movement Disorders Clinic, National Institute of Neurology and Neurosurgery, Mexico City, Mexico; ^2^Department of Neurosciences, University of Padova, Padua, Italy

**Keywords:** impulse control and compulsive behaviors, impulsivity, impulse control (pathology) disorders, related behavior, Parkinson's disease

Impulse control disorders (ICDs) are defined as a failure to resist a temptation, urge, or impulse leading to pursue certain reward-based activities or make poorly informed decisions without insight to the consequences of these repeated activities (1). First reports of ICDs in people with Parkinson's disease (PD) on dopaminergic replacement treatment began to appear in the early 2000s (2, 3). The first large study, the DOMINION Study, was published in 2010 establishing the relation of ICDs not only with dopaminergic treatment but with other demographic and clinical variables (4). Since then over 500 papers on ICD ranging from clinical features to neuroimaging and genetic risk factors have been published.

As editors of this special edition on Impulse Control Disorders, Impulsivity, and Related Behaviors in Parkinson's disease, we are pleased to present the collection of papers featured in this Research Topic.

The final collection is comprised of 11 high-quality papers including two minireviews, one review, and one perspective. In addition, three systematic reviews and four original research manuscripts complete this Research Topic.

In the review papers, Gatto and Aldinio shares a brief review on the definition and classification of ICD and their related behaviors, their prevalence, risk factors, clinical tools, neuroimaging, as well as their treatment. Garcia-Ruiz provides a comprehensive overview of ICD as a side effect of dopaminergic treatment but considering the possibility of an individual susceptibility mainly due to genetic factors. Finally, the author highlights another possible consequence of ICD manifested as enhanced creativity in persons with Parkinson without previous artistic abilities. De Micco et al. address the fact that not all persons with PD develop and ICD despite receiving dopaminergic treatment. They based their review on data derived for neuroimaging studies assessing dopaminergic signaling or reward processing. The authors conclude that there is evidence suggesting an increased dopaminergic firing in response to reward and that prospective multimodal imaging studies are still needed. Lastly, Eisinger et al. provide insight into some additional factors such as the role of country of residence, comorbidities, non-dopaminergic medications, and deep brain stimulation surgery. Overall, these set of paper give the reader a broad but comprehensive look at the current state of knowledge on ICDs and PD.

The systematic reviews include a meta-analysis of case-control studies by Molde et al. Along with confirmation of ICDs being s significantly associated with PD, being medically treated for PD and disease duration were the two variables associated with an increased risk of ICD. A meta-analysis from Martini et al. assessed PET or SPECT studies on dopaminergic neurotransmission in persons with PD and ICD. They conclude that persons with PD and ICD show lower dopaminergic transporter levels in the dorsal striatum and increased dopamine release in the ventral striatum when engaged in reward-related tasks. Another meta-analysis by Martini et al. evaluated measures of cognitive, affective, and motivational domains between PD subjects with and without ICDs. They found that ICD in PD is associated with poor reward-related decision-making, and neuropsychiatric symptoms such as depression, anxiety, and anhedonia.

Two original articles provide information derived from neuroimaging studies. Ruitenberg et al. assessed impulsivity by analyzing resting-state functional connectivity and structural MRI in subjects with and without ICDs. Their findings include reduced frontal-striatal connectivity and GPe volume were associated with more impulsivity. A different approach was taken by Zadeh et al. by shifting focus to alterations of white matter tract in drug-naïve subjects with PD and with ICDs using diffusion MRI connectometry. The authors report disrupted connectivity in the complex network of dynamic connections between cerebellum, basal ganglia, cortex, and its spinal projections.

Next, Erga et al. report some novel genetic data using whole-exome sequencing data from the Norwegian ParkWest study. Eleven SNPs were found to be associated with ICDs, with rs5326 in *DRD1* being the strongest risk factor, and rs702764 in *OPRK1* being associated with a decreased risk. Lastly, Martini et al. presents a cross sectional study comparing ICDs and related behaviors across people with PD with normal cognition, mild cognitive impairment and PD-related dementia. While frequency and severity of ICDs did not differ between groups, subjects with ICD showed more deficits within the attentive and executive domains in the mild cognitive impairment group.

We wish to thank all authors, and peer reviewers in the research featured. We hope that this collection of papers shed new light to the still expanding body of knowledge on ICDs in PD.

## Author Contributions

MR-V: conception, drafting the work, revising it and final approval of the version to be published. AA: conception, revising the work critically for important intellectual content and final approval of the version to be published.

### Conflict of Interest Statement

The authors declare that the research was conducted in the absence of any commercial or financial relationships that could be construed as a potential conflict of interest.
